# Supplementary material for: A phase I study of intra-anal artesunate (suppositories) to treat anal high-grade squamous intraepithelial lesions
Source: PLoS One. 2023 Dec 15;18(12):e0295647. doi: 10.1371/journal.pone.0295647 (PMC10723659; doi:10.1371/journal.pone.0295647)
Supplement: S1 Protocol — (DOC) [file pone.0295647.s004.doc]

**JHU Protocol #: IRB00090922**

**ClinicalTrials.gov Identifier:** NCT03100045

**TITLE:** **A Phase I Study of Intra-anally Administered Artesunate in Patients with High-Grade Anal Intraepithelial Neoplasia (AIN 2/3)**

**Corresponding Organization:** Johns Hopkins Hospital

**Principal Investigator:** Sandy Hwang Fang, M.D.

Johns Hopkins Hospital

Department of Surgery

Ravitch Division, Colon and Rectal Surgery

Blalock 618

600 N. Wolfe Street

Baltimore, MD 21287

T 410-955-7323

F 410-614-9866

[sfang7@jhmi.edu](mailto:sfang7@jhmi.edu)

**SITE : JOHNS HOPKINS UNIVERSITY**

**Co-Investigators**:

Cornelia Trimble, MD

Departments of Gynecology and Obstetrics, Oncology, and Pathology

Johns Hopkins University School of Medicine

Phipps 257

600 N. Wolfe Street

Baltimore, MD 21287

Phone: 410-502-0512

Fax: 443-769-1205

**Collaborators**:

Namandje N. Bumpus, PhD

Department of Medicine, Division of Clinical Pharmacology

Johns Hopkins School of Pharmacology and Molecular Sciences

725 N. Wolfe Street

Baltimore, MD 21205

Craig Hendrix, MD

Department of Medicine, Division of Clinical Pharmacology

Johns Hopkins School of Pharmacology and Molecular Sciences

Blalock 569

600 N. Wolfe Street

Baltimore, MD 21287

| **Statistician:**  Joseph Canner, MS  Johns Hopkins Surgery Center for Outcomes Research  Department of Surgery  Blalock 618  600 N. Wolfe Street  Baltimore, MD 21287 |  |
| --- | --- |

**Other Agent:**

**IND #: 134720**

**IND Sponsor:** Sandy Fang, MD

**Protocol Type / Version # / Version Date:**

Original / Version 8.1/ 3/16/2021

UNIVERSITY OF WISCONSIN

Site PI:

Evie H. Carchman, MD

K4/730 Clinical Science Center

University of Wisconsin Hospital and Clinics

600 Highland Avenue

Madison, WI 53792-7375

[carchman@surgery.wisc.edu](mailto:carchman@surgery.wisc.edu)

**Co-Investigators:**

Cristina B Geltzeiler, MD

K3/704 Clinical Science Center

University of Wisconsin Hospital and Clinics

600 Highland Avenue

Madison, WI 53792-7375

[geltzeiler@surgery.wisc.edu](mailto:geltzeiler@surgery.wisc.edu)

Elise H. Lawson, MD, MSHS

K4/736 Clinical Science Center

University of Wisconsin Hospital and Clinics

600 Highland Avenue

Madison, WI 53792-7375

[lawson@surgery.wisc.edu](mailto:lawson@surgery.wisc.edu)

PROTOCOL SYNOPSIS

| **Title of Study:** A Phase I Study of Intra-anally Administered Artesunate in Patients with High-Grade Anal Intraepithelial Neoplasia (AIN 2/3) |
| --- |
| **Investigators:**  **Principal Investigator**: Sandy Hwang Fang, MD, Department of Surgery, Johns Hopkins Hospital  **Co-Investigator**: Cornelia Trimble, MD, Department of Obstetrics and Gynecology, Department of Pathology, Johns Hopkins Hospital  **Collaborators**: Craig Hendrix, MD, Department of Medicine, Division of Clinical Pharmacology, Johns Hopkins School of Pharmacology and Molecular Sciences  Namandje N. Bumpus, PhD, Department of Medicine, Division of Clinical Pharmacology, Johns Hopkins School of Pharmacology and Molecular Sciences  **Statistician:** Joseph Canner, MS, Johns Hopkins Surgery Center for Outcomes Research |
| **Phase:** I |
| **Study Center:** Johns Hopkins Hospital |
| **Concept and Rationale:**  Up to 95% of anal cancers (anal squamous cell cancers, ASCC) are caused by human papillomavirus (HPV)[^1^](#_ENREF_1); however, current management of pre-cancerous lesions (anal intraepithelial neoplasia, AIN) consists of surgical excision or ablation and this does not treat the underlying cause of HPV infection. Recurrences are common; in HIV-seronegative persons treated with ablation, recurrence is as high as 50% within one year [^2^](#_ENREF_2). The morbidity of these destructive ablations is significant and includes anal stenosis and fecal incontinence.  Artesunate, formulated as a suppository, is WHO-approved for first-line treatment for acute malaria in children who are located in remote settings with limited access to healthcare [^3^](#_ENREF_3). Globally, Artesunate has been administered to 2 million people [^4^](#_ENREF_4), given orally, intramuscularly, intravenously, and as rectal suppositories, providing a wealth of safety information. Both Artesunate and dihydroartemisinin, an Artesunate metabolite, have been shown to be cytotoxic to epithelial cells expressing HPV16 E6 and/or E7, while having no effect on uninfected cells [^5^](#_ENREF_5). Both ASCC and AIN2/3 (high-grade anal intraepithelial neoplasia) are associated with functionally obligate expression of two viral proteins, E6 and E7. Epithelial cells that express either or both of these oncoproteins overexpress the transferrin receptor, and have increased levels of intracellular iron, compared to normal cells [^6^](#_ENREF_6). Artesunate contains an endoperoxide bridge that reacts with intracellular ferrous iron to generate free radicals, leading to cell death .  This observation raises the possibility of treating pre-invasive HPV disease (i.e., AIN2/3) with an effective anti-malarial drug, Artesunate, administered as an intra-anal suppository. The toxicity profile of this formulation is well-documented and includes dizziness, nausea, emesis, and abdominal pain. It should be noted that these symptoms are also commonly experienced in active malarial infection. Preliminary data from an ongoing study of intravaginal administration of Artesunate suppositories for cervical dysplasia has demonstrated mucosal irritation as the only adverse event.  This study investigates a novel non-surgical approach to the treatment of HPV-associated anal intraepithelial neoplasia, using topical Artesunate. |
| **Primary Objective(s):**  To evaluate the safety and tolerability of intra-anal administration of Artesunate, administered via suppository with 3 different schedules, in patients with high-grade anal intraepithelial neoplasia (AIN 2/3). |
| **Secondary Objective(s):**   - - 1. To measure the effect of intra-anal topical Artesunate administration determined by pathology, based on the regression of AIN2/3 at study weeks 16, 28, and 40. Regression defined as either AIN1 or no AIN lesion detected by HRA/biopsy and anal cytology.     2. To evaluate clearance of HPV as assessed by Hybrid Capture 2 DNA testing of cytologic specimens.     3. Collection patient specimens into tissue bank for future studies. |
| **Primary Endpoint(s):** Maximum safe dose of Artesunate. |
| **Secondary Endpoint(s):**   - - - - Tissue concentrations of Artesunate       - Change in pathology from AIN2/3 to AIN 1 or no evidence of AIN through the measurement of HPV genotyping.       - Tissue bank of specimens for future studies |
| **Study Design:**  This is a Phase I modified 3 + 3 design, in which the maximum tolerated dose (MTD) will be identified. The 3 + 3 dose escalation will consist of 6 dose levels in combination with variation in dosing schedules of the single-agent Artesunate. This design also allows for some possible intermediate doses to be examined if dose limiting toxicities (DLTs) occur and de-escalation is needed. An expansion cohort will occur at the MTD. Once the MTD is determined, then secondary outcomes will be evaluated. |
| **Number of Patients:** At Johns Hopkins Hospital, 6 new patients with the diagnosis of high-grade intraepithelial lesion (HSIL) are seen per month in our institution’s High-Resolution Anoscopy Clinic, which is our anal cancer screening clinic. Each year, over 72 new patients with the diagnosis of AIN 2/3 are seen. There is currently a 6-month waitlist for the High-Resolution Anoscopy Clinic. We anticipate that we will enroll 40% of our patients due to compliance and follow-up patterns studied in our HRA Clinic.  At the University of Wisconsin, on average 9 new patients with the diagnosis of high-grade anal intraepithelial neoplasia (HGAIN) are seen per month in our institution’s High-Resolution Anoscopy Clinic, which is our anal cancer screening clinic.  We anticipate the study duration to be 2 years. |
| **Main Criteria for Inclusion/Exclusion:**  Inclusion criteria:   - Age ≥ 18 years - Biopsy-confirmed high-grade anal dysplasia (AIN 2, AIN 3, HSIL) by HRA. This includes patients who are newly diagnosed with AIN 2/3 as well as those who have recurrent AIN 2/3 after medical therapy or surgical therapy. - Adequate contraceptive use for males and females of reproductive potential. - Female of childbearing potential: negative urine pregnancy test - Able to provide informed consent - Patients who have the ability to collaborate with planned follow-up (transportation, compliance history, etc.) - Patients who have and have not been immunized with the HPV vaccine. - Weight ≥50 kg.   Exclusion criteria:   - Age < 18 years - Diagnosis of low-grade anal dysplasia (AIN 1, LSIL) by HRA - Known anal, vulvar, cervical, or penile cancer - CD4 count < 200 at the time of consideration for entry into this study. Patients, whose CD4 counts drop below 200 at any timepoint in this study will be treated with the standard of care treatment arm of surgical ablation. - Unable to provide informed consent - Currently receiving systemic chemotherapy or radiation therapy for another cancer. - Patients who are on medical treatment with systemic immunosuppressants or steroids (e.g., active autoimmune disease) - Extensive anal condyloma precludes the ability for the clinician to visualize HSIL during HRA - Pregnant female - Weight < 50 kg. |
| **Intervention and Mode of Delivery:**  Artesunate suppositories will be administered transanally. Doses of escalation will be 200 mg, 400 mg, and 600 mg. Suppositories will be administered daily for 5 days. Five days constitutes 1 cycle. Up to 3 cycles will be administered at Weeks 0, 2, and 4. |
| **Duration of Intervention and Evaluation:**  Patients will undergo initial screening by HRA to evaluate candidates who have AIN 2/3. Week 0 starts when the patient starts their first cycle of Artesunate. Patients may take up to 3 cycles of Artesunate on Weeks 0, 2, and 4. HRA and biopsy will be performed at 3 month-intervals (Week 16, 28, 40), which is the standard of care for interval follow-up for AIN 2/3. |
| **Statistical Methods**:   1. **Definition of primary outcome/endpoint:** This is a Phase I modified 3 + 3 design, in which the MTD will be identified. 2. **Definition of secondary outcomes/endpoints:**  - Downgrade in AIN 2/3 to AIN 1 or no AIN - HPV genotype testing—optimally to undetectable levels of HPV at week 40 of follow. - Creating of tissue bank for future studies.   **Analytic plan for primary objective:** This is a Phase I modified 3 + 3 design, in which the MTD will be identified. Based on previous studies of rectally administered Artesunate for the treatment of malaria, the safety profile demonstrates a low probability of dose limiting toxicity in children, as well as adults. The initial dose will be a 200 mg suppository administered in cycles of 5 daily doses at weeks 0 and 2. Dose escalation will proceed with a 3 + 3 design with the safeguard of an integrated de-escalation schema. At the MTD, an expansion cohort of 12 patients will be included.  Note: Each cycle = 5 days   | **Dose Escalation Design** | | | | | | --- | --- | --- | --- | --- | | **Cohort** | **Dose (mg)** | **Number of Treatment Cycles** | **Designated Weeks of Artesunate Administration** | **Number of patients** | | **I** | 200 | 2 | 0, 2 | 3 | | **II** | 200 | 3 | 0, 2, 4 | 3 | | **III** | 400 | 2 | 0, 2 | 3 | | **IV** | 400 | 3 | 0, 2, 4 | 3 | | **V** | 600 | 2 | 0, 2 | 3 | | **VI** | 600 | 3 | 0, 2, 4 | 3 |   ***According to the standard of care clinical practice, a postoperative visit will be done 4 weeks after ablation, i.e. either on week 20 or on week 32   1. **Analytic plan for secondary objectives:**     1. For the dichotomous variables of regression of AIN 2/3, counts, percents and 95% confidence levels will be calculated.    2. HPV viral genotyping will be assessed to determine when HPV viral load will become undetectable.    3. Tissue bank of specimens for future studies. 2. **Sample size justification:** In this phase I dose escalation trial, 6 dose levels will be considered for a total sample size of 36-60 patients with a target dose limiting toxicity (DLT) of < 18%. |
| **Funding, Regulatory, and Feasibility Issues:**  Frantz Viral Therapeutics will supply the Artesunate suppositories at no cost. The suppository will be obtained from an off-site pharmacy (Buderer Drug Company).  Buderer Drug Company will ship suppositories to the Johns Hopkins Hospital Investigational Drug Pharmacy for each patient enrolled in the study. |
| **Patient Acceptability/Ethics and Consent Issues:** The issue that may limit patient or advocate acceptance would be compliance to follow-up, as patients will be on a strict schedule of up to 3 cycles of Artesunate suppository administration. Education of administration of the suppositories will be performed at the beginning of each cycle by the research nurse coordinator and a follow-up phone call at the end of each cycle of administration will determine any adverse events. Standard of care consists of surgical ablation and patients may opt to proceed with surgery at any time during this study. |

**Schema**


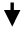

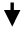

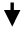

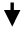


**Screening visit—HRA, anal pap, HPV genotyping**

**Week 0 (Cycle 1)**

Artesunate suppositories

A

**Week 2 (Cycle 2)**

Artesunate suppositories (Anoscopy)

**Week 4 (Cycle 3)**

Artesunate suppositories (Anoscopy )

**Week 16**

Assess for residual disease: HRA + biopsy + pap smear + HPV genotyping

Some improvement observed:

- Lesion size by office HRA
- AIN grade reduction (Biopsy, pap smear)

No improvement

Ablation in OR

**Week 28**

Assess for residual disease: HRA + biopsy + pap smear + HPV genotyping

No detection of AIN2/3 lesion by biopsy + anal pap smear

Detection of AIN2/3 lesion by biopsy + anal pap smear

Ablation in OR***

**Week 40**

Assess for relapsing disease: pap smear and if clinically indicated HRA + biopsy + HPV genotyping

**Week 6**

(Follow-up Anoscopy, HPV genotyping )

**TABLE OF CONTENTS**

[PROTOCOL SYNOPSIS 6](#__RefHeading___Toc37956931)

[1. OBJECTIVES 13](#__RefHeading___Toc37956932)

[1.1 Primary Objectives 13](#__RefHeading___Toc37956933)

[1.2 Secondary Objectives 13](#__RefHeading___Toc37956934)

[2. BACKGROUND 13](#__RefHeading___Toc37956935)

[2.1 HPV-associated malignancies of the anus 13](#__RefHeading___Toc37956936)

[2.2 Noninvasive topical therapy for anal dysplasia 14](#__RefHeading___Toc37956937)

[2.3 Artesunate 14](#__RefHeading___Toc37956938)

[2.4 Study Drug Rationale 15](#__RefHeading___Toc37956939)

[2.5 Study Drug Dosing Rationale 16](#__RefHeading___Toc37956940)

[3. PATIENT SELECTION 17](#__RefHeading___Toc37956941)

[3.1 Eligibility Criteria 17](#__RefHeading___Toc37956942)

[3.2 Exclusion Criteria 17](#__RefHeading___Toc37956943)

[3.3 Inclusion of Women and Minorities 18](#__RefHeading___Toc37956944)

[4. REGISTRATION PROCEDURES 18](#__RefHeading___Toc37956945)

[4.1 General Guidelines 18](#__RefHeading___Toc37956946)

[5. TREATMENT PLAN 18](#__RefHeading___Toc37956947)

[5.1 Agent Administration 18](#__RefHeading___Toc37956948)

[5.2 Definition of Dose-Limiting Toxicity 21](#__RefHeading___Toc37956949)

[5.3 Duration of Therapy 21](#__RefHeading___Toc37956950)

[5.4 Duration of Follow Up 22](#__RefHeading___Toc37956951)

[5.5 Criteria for Removal from Study Treatment 22](#__RefHeading___Toc37956952)

[6. ADVERSE EVENTS: LIST AND REPORTING REQUIREMENTS 22](#__RefHeading___Toc37956953)

[6.1 Definition of a Serious Adverse Event 23](#__RefHeading___Toc37956954)

[6.2 Adverse Event Characteristics 23](#__RefHeading___Toc37956955)

[6.3 Expedited Adverse Event Reporting 23](#__RefHeading___Toc37956956)

[6.4 AE Reporting 24](#__RefHeading___Toc37956957)

[6.5 Description of Study Medication 25](#__RefHeading___Toc37956958)

[6.6 Suppository Formulation 26](#__RefHeading___Toc37956961)

[6.7 Packaging and Labeling 26](#__RefHeading___Toc37956962)

[6.8 Product Storage and Stability 26](#__RefHeading___Toc37956963)

[6.9 Dosage and Administration 26](#__RefHeading___Toc37956964)

[6.10 Accountability Procedures for the Study Investigational Drugs 27](#__RefHeading___Toc37956965)

[6.11 Assessment of Subject Compliance with Self Administration of Artesunate Suppositories 27](#__RefHeading___Toc37956966)

[7. BIOMARKER, CORRELATIVE, AND SPECIAL STUDIES 28](#__RefHeading___Toc37956967)

[7.1 Collection of Specimens 30](#__RefHeading___Toc37956969)

[7.2 Pathology slides (Biopsy and resection tissue samples) 30](#__RefHeading___Toc37956970)

[7.3 Handling of Specimens 30](#__RefHeading___Toc37956971)

[7.4 Sites Performing Correlative Studies 30](#__RefHeading___Toc37956972)

[8. STUDY EVALUATION 30](#__RefHeading___Toc37956973)

[8.1 Description of Study Visits 30](#__RefHeading___Toc37956974)

[8.2 Visit intervals 31](#__RefHeading___Toc37956975)

[8.3 Examinations, Evaluations, and Procedures 31](#__RefHeading___Toc37956976)

[8.3.7 HIGH-RESOLUTION ANOSCOPY examination 32](#__RefHeading___Toc37956977)

[8.3.8 HRA, BIOPSY, AND SURGICAL ABLATION 32](#__RefHeading___Toc37956978)

[8.4 Compensation 33](#__RefHeading___Toc37956979)

[9. MEASUREMENT OF EFFECT 33](#__RefHeading___Toc37956980)

[10. DATA REPORTING / REGULATORY REQUIREMENTS 33](#__RefHeading___Toc37956981)

[10.1 Data Reporting 33](#__RefHeading___Toc37956982)

[11. Subject completion/dropout 34](#__RefHeading___Toc37956983)

[11.1 Definition of a Dropout 34](#__RefHeading___Toc37956984)

[11.2 Procedures for Handling Dropouts 34](#__RefHeading___Toc37956985)

[11.3 Reasons for Dropout 34](#__RefHeading___Toc37956986)

[12. CLINICAL MONITORING 35](#__RefHeading___Toc37956987)

[13. ETHICS AND REGULATORY CONSIDERATIONS 35](#__RefHeading___Toc37956988)

[14. STATISTICAL CONSIDERATIONS 37](#__RefHeading___Toc37956989)

[14.1 Study Design/Endpoints 37](#__RefHeading___Toc37956990)

[14.2 Sample Size/Accrual Rate 39](#__RefHeading___Toc37956991)

[14.3 Analysis of Primary and Secondary Endpoints 39](#__RefHeading___Toc37956992)

[15. REFERENCES 40](#__RefHeading___Toc37956993)

[APPENDIX A: STUDY CALENDAR 42](#__RefHeading___Toc37956994)

# OBJECTIVES

## Primary Objectives

- - 1. To evaluate the safety and tolerability of intra-anal administration of Artesunate, administered via suppository with 3 different schedules, in patients with high-grade anal intraepithelial neoplasia (AIN 2/3).]

## Secondary Objectives

- - 1. To measure the effect of intra-anal topical Artesunate administration determined by pathology, based on the regression of AIN2/3 at study weeks 16, 28, and 40. Regression defined as either AIN1 or no AIN lesion detected by HRA/biopsy and anal cytology.
    2. To evaluate the clearance of HPV as assessed by HPV genotype testing of cytologic specimens.

1.2.3 To create a tissue bank for future studies.

# BACKGROUND

## HPV-associated malignancies of the anus

Virtually all anal squamous cell cancers (ASCC) are caused by human papillomavirus (HPV). Exposure to HPV occurs with the onset of sexual activity. While most people clear their infection without intervention, and without sequelae, a subset does not. Persistent infection with a high-risk HPV type (most commonly types 16 and 18) is the most common cause of ASCC as well as anal precursor intraepithelial lesions, anal intraepithelial neoplasia (AIN2/3). Additional factors contribute to the development of ASCC, since viral integration into the host genome is necessary but not sufficient for the initiation and persistence of malignant transformation. Currently, there are no available treatments that can eradicate HPV infection. Successful prevention of ASCC in infected individuals is based on early detection of preinvasive lesions prior to the development of cancer.

Although preventative vaccines that protect against infection with HPV types 16 and 18 have been introduced for preteen and teenage girls and boys since 2006, the rates of vaccination have remained low in the United States. Recent data indicate that the rate of preventive vaccination in eligible girls, in 2012, was only 33%.[^9^](#_ENREF_9) The impact of HPV vaccination is yet to be determined, given the young age of vaccine administration and the long time interval in which anal cells transform through the anal dysplasia-carcinoma sequence.

Despite the availability of several, inexpensive, noninvasive screening strategies to detect precursor lesions, the incidence of HPV disease (ASCC and AIN 2/3) in patients remains high. In human immunodeficiency virus (HIV)-seropositive persons, the incidence of anal cancer has increased exponentially over the past few decades, making it one of the most common HIV-associated cancers.

The primary strategy to decrease disease burden in HPV-infected individuals is to intervene when premalignant anal disease, AIN2/3, is detected prior to the development of ASCC. All treatments for AIN2/3 are ablative and include electrocauterization/fulguration, hyfrecation, laser vaporization. Because they involve tissue destruction, all current therapeutic options have the potential for adverse sequelae, such as pain, fecal incontinence, and anal stenosis. Furthermore, these ablative techniques are not curative. Recurrences are common; in HIV-seronegative persons treated with ablation, recurrence is as high as 50% within one year of ablation.[^2^](#_ENREF_2) In HIV-positive individuals, recurrence rates are as high as 68%.[^10^](#_ENREF_10) An effective topical intervention that targets the underlying cause, HPV, would obviate the need for surgery and prevent progression to ASCC.

## Noninvasive topical therapy for anal dysplasia

Current available off-label topical treatment options include Imiquimod and 5-fluorouracil. Curative rates with these topical agents is poor, with complete reponse rates of 17% in patients using 5-fluorouracil and 24% patients using Imiquimod. Recurrence rates are high—58% in 5-fluorouracil users and 71% in Imiquimod users. Forty-three percent of imiquimod users and 27% in the 5-fluorouracil users experience grade 3-4 toxicities, leading to poor compliance rates.

The development of an effective topical therapy for preinvasive AIN2/3 lesions that could be self- administered would be a game-changing innovative treatment option for patients with preinvasive HPV disease. A topical treatment option would decrease adverse sequelae related to ablative procedures, decrease visits to specialized healthcare providers, and result in considerably decreased healthcare costs. In addition, in many cultures, HPV disease, carries a social stigma.[^12^](#_ENREF_12) The development of a topical therapy that could potentially be self-administered, would be empowering for patients with preinvasive lesions. A topical therapeutic also has the potential to obviate the need for surgery.

## Artesunate

Artesunate is a semisynthetic derivative of artemisinin, a plant compound extracted from the leaves of sweet wormwood, *Artemisia annua,* an herb used in Chinese traditional medicine for antimalarial treatment for over two thousand years, with marketed formulations since the late 1980s.[^13^](#_ENREF_13)

The safety profile of Artesunate and related compounds has been established based on millions of malaria patients, ranging from infants to adults, over decades.[^14^](#_ENREF_14) Artesunate has been shown to be safe and well-tolerated when administered orally, intravenously, intramuscularly, or intra- rectally, as a suppository.[^15-19^](#_ENREF_15) Reports submitted to the FDA by both the WHO and by Novartis summarize many of the safety studies (see Section 2.4) conducted with artemisinin and its derivatives, including artesunate.

Recently, Artesunate compounds have been shown to have antitumor activity against several human solid tumor cell lines, including breast, colon, ovarian, prostate, renal, and non-small cell lung cancer.[^18^](#_ENREF_18) Of particular relevance, artemisinin derivatives have also been shown to have a cytotoxic effect on HPV-immortalized epithelial cells.[^18^](#_ENREF_18)

Artemisinin and its derivatives, including Artesunate, have been shown to decrease cell proliferation, reduce angiogenesis and trigger apoptosis in cancer cells. Although the mechanisms of action are not completely understood, in human solid cancers, sensitivity to Artesunate has been reported to be associated with expression of angiogenesis-related gene transcripts.[^23^](#_ENREF_23) Tumor susceptibility has also been reported to be correlated with cell surface overexpression of transferrin receptor [^24^](#_ENREF_24) and with intracellular ferrous iron.[^6^](#_ENREF_6) It has been demonstrated that artemisinin and its derivatives contain an endoperoxide bridge that reacts with intracellular ferrous iron to generate free radicals, leading to cell death.[^15^](#_ENREF_15)

## Study Drug Rationale

As with cervical squamous cell cancer and cervical intraepithelial neoplasia, both ASCC and AIN2/3 are associated with functionally obligate expression of two viral proteins, E6 and E7.[^25^](#_ENREF_25) Epithelial cells that express either or both of these oncoproteins also overexpress the transferrin receptor, and have increased levels of intracellular iron, compared to normal cells.

Because cervical squamous cell cancers and their precursors, intraepithelial lesions, overexpress the transferrin receptor,[^23^](#_ENREF_23) these observations prompted a subsequent study of the cytotoxic effect of dihydroartemisinin (DHA), the bioactive form of Artesunate, on papillomavirus-expressing epithelial cells.[^5^](#_ENREF_5) *In vitro* studies demonstrated that, while DHA had little effect on normal cervical epithelial cells, it had a significant cytotoxic effect on HPV-immortalized cervical cells.[^5^](#_ENREF_5) In addition, formulated as a local treatment (2.22 mg DHA dissolve in 100 µl dimethylsulfoxide) in an oral mucosal canine nonclinical model which has 100% known tumor growth rate with HPV-infection, DHA has been reported to inhibit papillomavirus-induced tumor formation. In addition, tumor-negative dogs developed antibodies against the HPV L1 capsid protein.[^5^](#_ENREF_5)

Together, these findings suggest that topical administration of an Artesunate compound may provide therapeutic benefit for intraepithelial HPV disease. Given the unmet need for a local treatment for AIN2/3 lesions and that Artesunate has demonstrated a favorable safety profile for decades in both children and adults, we designed a prospective, dose-escalation, Phase 1 study of an Artesunate suppository, administered intra-anally, to non-invasively treat AIN2/3 lesions.

## Study Drug Dosing Rationale

- - 1. **Single Dose Toxicity**

The LD_50_ (lethal dose that kills 50 percent of the test sample) of Artesunate, given as intravenous and intramuscular injections to mice were determined as 699 mg/kg and 475 mg/kg, respectively. Reduced appetite and emesis were the only clinical symptoms reported with a rising dosing schedule in dogs of 37 mg/kg on day 1, 75 mg/kg on day 2, and 150/kg on day 3. Maximal tolerated doses in monkeys were 60 mg/kg and lethal doses were found to be 160 mg/kg. The adverse effects observed were central nervous system effects (depression, unstable gait, tremor, and convulsion) and respiratory depression. In the oral formulation, dogs have tolerated doses of up to 120 mg/kg with some liver damage at this concentration or higher.[^14^](#_ENREF_14)

- - 1. **Repeated Dose Toxicity**

In repeated dose-toxicity studies, intravenous administration of artesunate into canines, at doses of 10 and 40 mg/kg for 14 days, did not produce clinical symptoms or significant hematological, biochemical, or histopathological changes. Monkeys who were given daily doses of 10 and 32 mg/kg daily intravenous injections for 14 days did not experience adverse effects.[^14^](#_ENREF_14)

- - 1. **Organ Toxicity**

No artemisinin-induced embryotoxicity has been reported in humans.[^26^](#_ENREF_26) Neurotoxicity-associated with artemisinins appears to be not only dependent on dose but also on the route of administration and the type of artemisinin derivative. Water-soluble artesunate shows markedly less neurotoxicity in laboratory animals than oil-soluble arteether and artemether.[^26^](#_ENREF_26) In mice, neurotoxic effects of artesunate in a 28-day oral dosing schedule were observed at a dose of >200 mg/day.[^27^](#_ENREF_27)

- - 1. **Dosing rationale**

In summary, local tissue application in canine models infected with oral mucosal HPV-derived tumors of a dose of 2.22 mg resulted in an absence of tumor formation. This dosage is significantly less than the first-line 10 mg/kg anti-malarial rectal suppository dosing administered to young children and the 200 mg suppository available for adults (4 mg/kg for a 50-kg individual). No serious adverse events are anticipated at the proposed dose escalation levels of 200 mg (4 mg/kg for a 50-kg adult), 400 mg (8 mg/kg for a 50-kg adult), and 600 mg (12 mg/kg for a 50-kg adult). A five-day daily dosing schedule is selected based on the clinical toxicology and pharmacology literature of anti-malarial and cancer trials.

We anticipate that this Phase I dose escalation trial of intra-anal suppository administration will achieve local tissue drug concentrations adequate to achieve cytotoxicity of HPV-infected dysplastic cells.

# PATIENT SELECTION

## Eligibility Criteria

- Age ≥ 18 years.
- Biopsy-confirmed high-grade anal dysplasia (AIN 2, AIN 3, HSIL) by HRA. This includes patients who are newly diagnosed with AIN 2/3 as well as those who have recurrent AIN 2/3 after medical therapy or surgical therapy.
- Female of childbearing potential: negative urine pregnancy test.
- Adequate contraceptive use for males and females of reproductive potential.
- Able to provide informed consent.
- Patients who have the ability to collaborate with planned follow-up (transportation, compliance history, etc.).
- Patient who have and have not been immunized with the HPV vaccine.
- Weight ≥ 50 kg.
- Life expectancy > 2 years.

## Exclusion Criteria

- Age < 18 years
- Diagnosis of low-grade anal dysplasia (AIN 1, LSIL) by HRA
- Known anal, vulvar, cervical, or penile cancer
- CD4 count < 200 at the time of consideration for entry into this study. Patients, whose CD4 counts drop below 200 at any timepoint in this study, will be pulled to the standard of care treatment arm of surgical ablation.
- Unable to provide informed consent
- Currently receiving systemic chemotherapy or radiation therapy for another cancer.
- Patients who are on medical treatment with systemic immunosuppressants or steroids (e.g., active autoimmune disease)
- Extensive anal condyloma precludes the ability for the clinician to visualize HSIL during HRA
- Pregnant female
- Weight < 50 kg.

## Inclusion of Women and Minorities

NIH policy requires that women and members of minority groups and their subpopulations be included in all NIH-supported biomedical and behavioral research projects involving NIH-defined clinical research unless a clear and compelling rationale and justification establishes to the satisfaction of the funding Institute & Center (IC) Director that inclusion is inappropriate with respect to the health of the subjects or the purpose of the research. Exclusion under other circumstances must be designated by the Director, NIH, upon the recommendation of an IC Director based on a compelling rationale and justification. Cost is not an acceptable reason for exclusion except when the study would duplicate data from other sources. Women of childbearing potential should not be routinely excluded from participation in clinical research. Please see <http://grants.nih.gov/grants/funding/phs398/phs398.pdf>.

This clinical trial will not exclude potential subjects from participating in this or any study solely on the basis of ethnic origin or socioeconomic status. Every attempt will be made to enter all eligible patients in this protocol to address the study objectives in a population representative of patients with solid organ tumors treated by any of the participating institutions. (See Planned Enrollment Table in Section 14.4)

# REGISTRATION PROCEDURES

## General Guidelines

Following registration, patients should begin protocol treatment within 4 weeks. Any issues that would cause treatment delays should be discussed with the site Principal Investigator. If a patient does not receive protocol therapy following registration, the patient’s registration on the study may be canceled. The Study Coordinator should be notified of cancellations as soon as possible.

# TREATMENT PLAN

## Agent Administration

Treatment will be administered either on an outpatient basis or remotely, as a telemedicine visit. Reported adverse events and potential risks are described in Section 7. Appropriate dose modifications are described in Section 6. No investigational or commercial agents or therapies other than those described below may be administered with the intent to treat the patient’s disease process.

| **Dose Escalation Design** | | | | |
| --- | --- | --- | --- | --- |
| **Cohort** | **Dose (mg)** | **Number of Treatment Cycles** | **Designated Weeks of Artesunate Administration** | **Number Subjects** |
| **I** | 200 | 2 | 0, 2 | 3 |
| **II** | 200 | 3 | 0, 2, 4 | 3 |
| **III** | 400 | 2 | 0, 2 | 3 |
| **IV** | 400 | 3 | 0, 2, 4 | 3 |
| **V** | 600 | 2 | 0, 2 | 3 |
| **VI** | 600 | 3 | 0, 2, 4 | 3 |

***Note: Each cycle = 5 days

Artesunate suppositories will be administered intra-anally on days 1 to 5, and the dosing schedules will vary between 2 and 3 cycles. A dose-limiting toxicity for the Artesunate suppository is defined as any Grade 2 or greater toxicities in any organ system (or Grade 3 or greater toxicities), as delineated in CTCv4.0. Patients will be continued on treatment until disease progression to cancer, unacceptable toxicity, death, pregnancy, withdrawal of consent, or study termination.

The MTD will be determined utilizing a modified 3 + 3 design. The 3 + 3 dose escalation will consist of 6 dose levels in combination with variation in dosing schedules the single agent Artesunate, with additional de-escalation steps. An expansion cohort of 12 patients will be included at the MTD. Once MTD is found, then secondary outcomes will be evaluated.

**Cohort I**:

**200 mg** x 5 daily doses/cycle.

**2 cycles** administered at weeks 0, 2

**Cohort III**:

**400 mg** x 5 daily doses/cycle.

**2 cycles** administered at weeks 0, 2

**Cohort IV**:

**400 mg** x 5 daily doses/cycle.

**3 cycles** administered at weeks 0, 2, 4

**De-escalate** to **200 mg** x 5 daily doses/cycle.

**1 cycle** administered at week 0

**De-escalate** to **400 mg** x 5 daily doses/cycle.

**1 cycle** administered at week 0

**Cohort II**:

**200 mg** x 5 daily doses/cycle.

**3 cycles** administered at weeks 0, 2, 4

**Cohort VI**:

**600 mg** x 5 daily doses/cycle.

**3 cycles** administered at weeks 0, 2, 4

**Cohort V**:

**600 mg** x 5 daily doses/cycle.

**2 cycles** administered at weeks 0, 2

**De-escalate** to **600 mg** x 5 daily doses/cycle.

**1 cycle** administered at week 0

**At MTD, expansion cohort**, n = 12.

De-escalate

De-escalate

De-escalate

De-escalate

## Definition of Dose-Limiting Toxicity

A dose-limiting toxicity (DLT) is defined as any drug-related Grade 2 (moderate; minimal, local, or noninvasive intervention indicated; limiting age appropriate instrumental ADL) or greater drug-related toxicities in any organ system (or Grade 3 or greater toxicities–severe or medically significant but not immediately life-threatening; hospitalization or prolongation of hospitalization indicated; disabling; limiting self care ADL**) in any organ system (including gastrointestinal, dermatologic, and neurologic), as delineated in Common Terminology Criteria for Adverse Events v4.0 (CTCAE).

This above definition of dose-limiting toxicity applies to the drug that is being studied—the artesunate suppository. Post-procedural complications related to the HRA procedure are not dose limiting.

The dose limiting toxicity determining period for the study will extend to 1 week after the last cycle of artesunate suppository. Thus, for the 2 cycles, the DLT determining period will extend to 3 weeks; for 3 cycles, it will extend to 5 weeks.

Beyond the DLT determining period, if patients in a specific cohort have complete resolution of their high-grade dysplastic lesions, then the plan will be to stop at this dosing cycle and frequency and move into the 12-patient expansion cohort.

Management and dose modifications associated with the above adverse events are outlined in Section 6.

Dose escalation will proceed within each cohort according to the modified 3+ 3 design with de-escalation schema and an expansion cohort at MTD. Dose-limiting toxicity (DLT) is defined as above.

The specific details are as follows,

The 3 + 3 dose escalation will consist of 6 dose levels in combination with variation in dosing schedules the single agent Artesunate, with additional de-escalation steps. An expansion cohort of 12 patients will be included at the MTD. Once MTD is found, then secondary outcomes will be evaluated. In MTD expanded patient cohort, anal intraepithelial neoplasia (AIN) regression from AIN 2/3 to low-grade anal intraepithelial neoplasia (AIN 1) or no residual AIN will be assessed through subsequent HRA evaluation. Cytology specimens will be tested through quantitative PCR for HPV viral load during initial and subsequent HRA evaluations. A tissue bank will be created for future studies.

## Duration of Therapy

In the absence of treatment delays due to adverse event(s), treatment may continue for the 40-week research study period or until one of the following criteria applies:

- Disease progression to cancer
- Intercurrent illness that prevents further administration of treatment
- Unacceptable adverse event(s)Patient decides to withdraw from the study
- General or specific changes in the patient’s condition render the patient unacceptable for further treatment in the judgment of the investigator.

## Duration of Follow Up

Patients will be followed for a total of 40 weeks from the first day of administration of the study drug or until death, whichever occurs first. Patients removed from study treatment for unacceptable adverse event(s) will be followed until resolution or stabilization of the adverse event.

Please see the schematic diagram for treatment follow-up as outlined in the Protocol Synopsis section.

## Criteria for Removal from Study Treatment

Patients will be removed from study treatment when any of the criteria listed in Section 5.3 applies. The reason for study removal and the date the patient was removed must be documented in the Case Report Form.

#

# ADVERSE EVENTS: LIST AND REPORTING REQUIREMENTS

Because this is a pilot study evaluating tolerability of a new intra-anal treatment, the stopping rule for safety is the presence of drug-related Grade 3 toxicity in any organ system (including gastrointestinal, dermatologic, and neurologic toxicities) delineated in CTCAEv4.0. In the event of two identical unexpected treatment-related Grade 2 or greater toxicities in any organ system ( or other Grade 3 or greater toxicities), accrual will be suspended pending further review.

Subjects who become pregnant during study will be required to discontinue drug. Although not considered an adverse event, pregnancy will be reported in the same way. Whenever possible, a pregnancy should be followed to term, and the status of mother and child reported. The Pregnancy Notification form will be sent to the principal investigator (Dr. Fang) within 48 hours of discovery. Whenever possible, the Follow-up Pregnancy Outcome Form will be filled after delivery.

Finally, if there is evidence of disease progression at the interval visit regardless whether the progression may or may not be associated with Artesunate, patients will undergo HRA biopsy and ablation at that point.

## Definition of a Serious Adverse Event

A serious adverse event or reaction is any untoward medical occurrence that at any dose:

- - - results in death
    - is life-threatening (an event in which the subject is at risk of death at the time of the event; it does not refer to an event which hypothetically might have caused death if it were more serious)
    - requires inpatient hospitalization or prolongation of existing hospitalization
    - results in persistent or significant disability or incapacity
    - is a birth defect/congenital anomaly

## Adverse Event Characteristics

- **CTCAE term (AE description) and grade:** The descriptions and grading scales found in the revised NCI Common Terminology Criteria for Adverse Events (CTCAE) version 4.0 will be utilized for AE reporting. All appropriate treatment areas should have access to a copy of the CTCAE version 4.0. A copy of the CTCAE version 4.0 can be downloaded from the CTEP web site <http://ctep.cancer.gov/protocolDevelopment/electronic_applications/ctc.htm>.
- **Attribution** of the AE:
  - Definite – The AE *is clearly related* to the study treatment.
  - Probable – The AE *is likely related* to the study treatment.
  - Possible – The AE *may be related* to the study treatment.
  - Unlikely – The AE *is doubtfully related* to the study treatment.
  - Unrelated – The AE *is clearly NOT related* to the study treatment.

## Expedited Adverse Event Reporting

The severity of each adverse event must be assessed using the NCI Common Terminology Criteria for Adverse Events, v4.0 (CTCAE 4.0).

All adverse events that occur from the time of study drug administration through 30 days after the final treatment will be recorded. Duration, severity, treatments administered and outcome for each adverse event will be recorded on the CRF (case report form) for the following visit. Drug related adverse events will be followed until resolved, until no further improvement is expected, until a non-study antitumor therapy is initiated, or for 30 days after last study treatment, whichever comes first (with the exception of neuropathy, which must be followed until resolution to toxicity grade 1 or until stable for 60 days).

Adverse events beginning 30 days after the last treatment that the investigator considers to be related to study treatment will be reported to the appropriate contact person at any time such events occur.

Conditions that were present at the study start and that worsen during the study should be reported as beginning on the date the event worsened, not the date it began pre-study. Event text may include the word “worsened” or “exacerbated.”

Conditions which were recorded as “intermittent” at the study start which occur during the study must be reported if they are more frequent or of greater severity.

**Phase 1 and Early Phase 2 Studies: Expedited Reporting Requirements for Adverse Events that Occur on Studies under an IND/IDE within 30 Days of the Last Administration of the Investigational Agent/Intervention** 1, 2

| **FDA REPORTING REQUIREMENTS FOR SERIOUS ADVERSE EVENTS (21 CFR Part 312)**  **NOTE:** Investigators **MUST** immediately report to the sponsor (NCI) **ANY** Serious Adverse Events, whether or not they are considered related to the investigational agent(s)/intervention (21 CFR 312.64)  An adverse event is considered serious if it results in **ANY** of the following outcomes:   1. Death 2. A life-threatening adverse event 3. An adverse event that results in inpatient hospitalization or prolongation of existing hospitalization for ≥ 24 hours 4. A persistent or significant incapacity or substantial disruption of the ability to conduct normal life functions 5. A congenital anomaly/birth defect. 6. Important Medical Events (IME) that may not result in death, be life threatening, or require hospitalization may be considered serious when, based upon medical judgment, they may jeopardize the patient or subject and may require medical or surgical intervention to prevent one of the outcomes listed in this definition. (FDA, 21 CFR 312.32; ICH E2A and ICH E6). | | |
| --- | --- | --- |
| **ALL SERIOUS** adverse events that meet the above criteria MUST be immediately reported to the NCI via electronic submission within the timeframes detailed in the table below. | | |
| **Hospitalization** | **Grade 3-5**  **Timeframes** |  |
| Resulting in Hospitalization  ≥ 24 hrs | 24-Hour 5 Calendar Days |  |
| Not resulting in Hospitalization  ≥ 24 hrs |  |
| **NOTE:** Protocol specific exceptions to expedited reporting of serious adverse events are found in the Specific Protocol Exceptions to Expedited Reporting (SPEER) portion of the CAEPR.  **Expedited AE reporting timelines are defined as:**   - “24-Hour; 5 Calendar Days” – The AE must initially be submitted electronically within 24 hours of learning of the AE, followed by a complete expedited report within 5 calendar days of the initial 24-hour report. | | |
| ^1^Serious adverse events that occur more than 30 days after the last administration of investigational agent/intervention and have an attribution of possible, probable, or definite require reporting as follows:  **Expedited 24-hour notification followed by complete report within 5 calendar days for:**   - All Grade 3, 4, and Grade 5 Aes | | |

## AE Reporting

Expected Aes will be reported to the JHM IRB and reviewed at the time of the annual review. The University of Wisconsin team will enter all expected adverse events into the REDCap database within 2 weeks. The JHU team will compile all Adverse Events from both institutions for the annual review by the JHM IRB. Any drug-related SAEs or multiple occurrences of an AE determined to be an unanticipated problem will be reported by both institutions to the Johns Hopkins Institutional Review Board and the Johns Hopkins Cancer Center Clinical Research Committee. Drug-related SAE’s will be reported to all regulatory agencies by the study PI (Dr. Fang) within 7 working days. Any drug-related SAEs reported by a study participant at the University of Wisconsin will be reported to the JHU PI within 3 business days, in order to allow sufficient time for the expedited reporting by the JHU PI. Unanticipated or unexpected AE is defined as any AE not listed in available sources including the labeling, the Investigator’s Brochure, or this protocol.

PHARMACEUTICAL INFORMATION

## Description of Study Medication

## Study Medication: Suppository Formulation of Artesunate

## Drug Substance

Artesunate is a derivative of artemisinin, which is the active principle of the Chinese medicinal herb *Artemisia annua*.

Nomenclature Artesunate, Artesunic acid

Chemical Name [3*R*, 5a*S*,6*R*,8a*S*,9*R*,10*S*,12*R*,12a*R*)-3,6,9-

trimethyldecahydro-3,12-epoxypyrano[4,3-*j*]-1,2- benzodioxepin-10-ol, hydrogen succinate;

CAS Reg. No. 88495-63-0


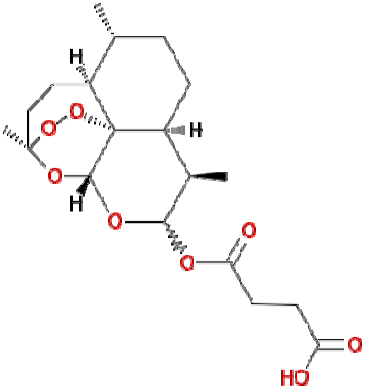


General Properties

Molecular Formula C19H28O8

Molecular Weight 384.4 g/mol

Physical Description A fine, white crystalline powder

Solubility Very slightly in water; very soluble in dichloromethane; freely soluble in ethanol (~ 750 g/l) and acetone

Melting Point 132-135ºC

Optical Rotation R; [α] D 20 °C = +2.5° to +3.5° in a 10mg/mL in

dichloromethane

pH 3.5-4.5 in an aqueous suspension containing 10 mg/g

## Suppository Formulation

Artesunate suppositories will be produced under controlled environmental conditions as prescribed in USP Chapter 795. Artesunate will be added and dissolved into a molten fatty acid base. The mixture will then be transferred into disposable pre-formed suppository molds to cool into solid suppositories.

Clinical trial supplies will be produced by Buderer Drug Co. in Perrysburg, Ohio. Buderer maintains compliance with USP Chapter 795 (Pharmaceutical Compounding – Nonsterile Preparations) and USP Chapter 797 (Pharmaceutical Compounding – Sterile Preparations), which establish practice standards and outline the responsibility of the compounder, selection and appropriate sources of ingredients, quality control, and considerations regarding the stability of compounded preparations. Two additional USP informational chapters are in effect, including, USP Chapter 1075 – Good Compounding Practices, and USP Chapter 1160 – Pharmaceutical Calculations in Prescription Compounding.

## Packaging and Labeling

Suppositories for dispensing will be packaged in standard prescription use plastic or cardboard containers. Proper labeling will be applied per state regulations.

## Product Storage and Stability

The suppositories may be stored at controlled room temperature. Suppositories for dispensing will be packaged in plastic or cardboard container, and kept in their plastic mold until administration.

USP 795 designates a beyond use date for this preparation not longer than 6 months. Potency testing will be obtained to validate the preparation process.

## Dosage and Administration

the first dosing visit (week 0) will be an in-person visit, in which tthe study physician or nurse will administer the first of five daily intra-anal suppositories for each enrolled subject at the study site. The subjects will be trained by the physician or nurse on the insertion of artesunate suppositories transanally during that visit, and instructed to administer four subsequent daily doses at home. All subsequent dosing visits (weeks 2 and 4) will be performed as telemedicine visits. For telemedicine visits, the physicians will review the symptom diaries and patients will be instructed about suppository administration over the phone. Within 5 days of the last day of the cycle(s), patients will receive a telephone call to assess for any AE. If patients do not experience any symptoms or AEs after suppository administration, then the subsequent dosing visits will be telemedicine visits. However, if patients experience AEs, then the subsequent dosing visits will be in-person visits, at which time the physician will perform an anoscopy.

The first 3 study subjects (healthy volunteers with AIN2/3) will receive five (5) daily suppositories that contain 200 mg Artesunate for a total of 2 cycles at weeks 0 and 2. The 200 mg dose (or ≤ 4 mg/kg for subjects weighing 50 kg at minimum) is considerably lower than that used when the anal suppository is intended to deliver a systemic dose of drug for malaria – the 10 mg/kg (50 mg) suppository is recommended by WHO for pediatric care. If no severe local irritation or systemic toxicities are observed in the first 3 patients at 200 mg, the dosage will escalate to 3 cycles of 200 mg per day. Doses of escalation will be 200 mg, 400 mg, and 600 mg. Suppositories will be administered daily for 5 days. Five days constitutes 1 cycle. Up to 3 cycles will be administered at Weeks 0, 2, and 4. Please see section 5 for dose escalation schema.

## Accountability Procedures for the Study Investigational Drugs

Clinical investigators will issue a prescription for each enrolled patient per USP 1168 and State and Federal Law. Buderer Drug Co. will schedule, prepare, package, label and ship clinical trial materials to participating facilities.

The study drug will be kept in the institutional Investigational Drug Pharmacy. Upon receipt of drug orders, and documentation of negative pregnancy test (for women of childbearing age), the pharmacist will dispense enough suppositories for a full cycle (5 days). When dosing visits are done as telemedicine, the Institutional Pharmacy will ship the suppositories to the patient’s home, per institutional guidelines.

## Assessment of Subject Compliance with Self Administration of Artesunate Suppositories

Eligible patients will have clinic or telemedicine visits scheduled at weeks 0, 2, and 4 for a urine pregnancy test (if female) and dosing. Once documentation of a negative pregnancy test is obtained (women of childbearing potential only), the patient will receive 5 doses of Artesunate for the respective treatment cycle. They will receive instructions on how to apply the suppositories and will apply the first dose of Artesunate during this visit. At each dosing visit, the study team will confirm contact information where they can be reached the following week. At dosing visits 2 and 3, if done in person, patients will have an anoscopy to assess the dentate line, and document any adverse events. For telemedicine visits, the investigator and/or study coordinator will review the Symptom Diary with the patient anddocument AEs. The Investigator will grade and determine causality to the study medication. Patients will have an in-person additional anoscopy and HPV genotyping at week 6 (+/- 1 week).

Patients will be monitored throughout the study for both known potential risks and for unexpected risks. During office visits, patients will be monitored specifically for any signs of:

- - - Symptoms of itching, burning, pain, warmth, or tenesmus
    - Signs of skin breakdown including erythema, blistering, desquamation, ulceration or bleeding
    - Neurologic symptoms
    - Unexpected adverse events

Subjects will be given diary cards to record local and general signs and symptoms most likely to occur between office visits. The diary cards will be collected, signs and symptoms verified, and inserted into the CRF at each subsequent visit.

# BIOMARKER, CORRELATIVE, AND SPECIAL STUDIES

- Downgrade in AIN 2/3 to AIN 1 or no AIN by HRA evaluation
- Decrease in HPV genotype testing by PCR of anal pap test sample—optimally to undetectable levels of HPV at week 40 of follow-up.
- Creation of tissue bank of specimens obtained from patients.

Please note that the highlighted tests above are depicted in the flow diagram and timeline in the schematic diagram on the next page.

##
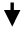

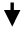

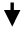


**Screening visit—HRA, anal pap, HPV genotyping**

**Week 0 (Cycle 1)**

Artesunate suppositories

A

**Week 2 (Cycle 2)**

Artesunate suppositories (Anoscopy)

**Week 4 (Cycle 3)**

Artesunate suppositories (Anoscopy )

**Week 16**

Assess for residual disease: HRA + biopsy + pap smear + HPV genotyping

Some improvement observed:

- Lesion size by office HRA
- AIN grade reduction (Biopsy, pap smear)

No improvement

Ablation in OR

**Week 28**

Assess for residual disease: HRA + biopsy + pap smear + HPV genotyping

No detection of AIN2/3 lesion by biopsy + anal pap smear

Detection of AIN2/3 lesion by biopsy + anal pap smear

Ablation in OR

**Week 40**

Assess for relapsing disease: pap smear and if clinically indicated HRA + biopsy + HPV genotyping

**Week 6**

(Follow-up Anoscopy )

##

## Collection of Specimens

Anal brushing tests (a.k.a. cytology brushings) will be obtained for both standard of care cytopathological evaluation and also to create a tissue bank for future analysis studies.

## Pathology slides (Biopsy and resection tissue samples)

Representative diagnostic hematoxylin and eosin slides cut from FFPE blocks of  diagnostic biopsy at study entry and post-artesunate biopsy and/or therapeutic resection/ablation will be sent by participating sites to JHU for centralized adjudication by an independent pathologist, blinded to outcomes.  We will evaluate the histologic ‘context’ in subject-matched tissue specimens obtained before, during, and after treatment. Whenever possible, additional recuts of the paraffin-embedded blocks will be sent to JHU for additional staining and analyses.

## Handling of Specimens

Regular anal pap testing and HRA biopsies will be submitted to Pathology as per standard hospital protocol to evaluate for pathological diagnosis. Samples for HPV genotyping will be sent to Atila Biosystems. Anal brushes and blood specimens will be banked in Dr. Trimble’s lab at Johns Hopkins. Anal brushes and blood specimens collected at the University of Wisconsin will be processed and stored locally and shipped to JHU in batch.

## Sites Performing Correlative Studies

Johns Hopkins Hospital

Pathology Laboratories – JHU, UWM

Atila Biosystems

Department of Clinical Pharmacology

# STUDY eVALUATION

Baseline evaluations are to be conducted within 4 weeks prior to start of protocol therapy. In the event that the patient’s condition is deteriorating, laboratory evaluations at the discretion of the physician should be performed within 48 hours prior to initiation of the next cycle of therapy.

Please refer to Study Calendar, Appendix A.

## Description of Study Visits

All clinically relevant data collected during the study procedures must be recorded first in the patient file. These data will then be transcribed into the CRF. All visits will be outpatient visits.

At the initial screening visit, in addition to undergoing a standard of care high-resolution anoscopy evaluation, we will obtain anal cytology brush specimens for HPV genotyping, Subjects with biopsy-confirmed AIN2/3 will be enrolled into treatment cohorts as previously described (see table). Subjects who underwent HRA with biopsy and pap smear within the 3 months preceding the screening visit will not need an additional biopsy and anal pap smear for study entry.

## Visit intervals

Study visits and study procedures will be carried out within the timeframe outlined below.

Table 1**: Visit/procedures timeline**

| **Screening visit** | **Biopsy diagnosis within the 3 months prior to the visit may be accepted for study entry**  **HIV testing and CD4 counts within the 6 months prior to study entry may be accepted for study entry** |
| --- | --- |
| **Week 0** | 4 weeks +/- 2 weeks after the screening visit  Questionnaire/phone f/u 2-7 days after last day of dosing |
| **Week 2 – may be done as televisit** | 2 weeks +/- 1 week  Questionnaire/phone f/u 2-7 days after last day of dosing |
| **Week 4 – may be done as televisit** | 4 weeks +/- 1 week  Questionnaire/phone f/u 2-7 days after last day of dosing |
| **Week 6** | 6 weeks +/- 1 week |
| **Week 16** | 16 weeks +/- 2 weeks |
| **Week 28** | 28 weeks +/- 2 weeks |
| **Week 40** | 40 weeks +/- 2 weeks |
| **Excision (if needed)** | Can take place any time after week 16 HRA |

## Examinations, Evaluations, and Procedures

**8.3.1 Vital signs**

Sitting blood pressure and heart rate will be measured both prior to each procedure and drug administration. Additional investigation may be added at the discretion of the investigator.

**8.3.2 Demographics/Medical history**

The patient’s age, weight, height, and ethnicity will be ascertained at the screening visit. Information will be collected on the patient’s past significant medical history including tobacco exposure, sexual practices, HIV status, contraceptive use, total and current number of partners, and current medical status.

**8.3.3 History of HPV-related disease**

The following information will be noted at the screening visit:

- Month/year of detection of AIN lesions, cervical, or vulvar lesions.
- Details of previous therapies.

**8.3.4 HIV test**

If positive HIV test, then will obtain CD4 count, if the patient has not had one within the last 6 months. Those patients with a CD4 count > 200 will be included in this study.

**8.3.5 Urine sample**

A urine pregnancy test for HCG will be performed in women of childbearing potential prior to each suppository administration.

**8.3.6 ANOSCOPY**

Anoscopy will be performed at the week 2 and week 4 dosing visits (if dosing visits take place as in-person visits) and/or within 2 week after the last administration of the artesunate suppository to evaluate for mucosal irritation from suppository administration.

# 8.3.7 HIGH-RESOLUTION ANOSCOPY examination

Patients will undergo serial HRA examinations; at the screening visit, and at weeks 16, 28, and 40. Exfoliated cell samples will be obtained using at the time of HRA, for anal cytology, HPV typing, and for tissue bank storage. Anoscopy will be performed and the anal canal will be visualized with a Zeiss colposcope. Serial applications of 5% acetic acid followed by Lugol’s solution into the anal canal will be analyzed by an anoscope to identify vascular abnormalities. Biopsies will be obtained with an endoscopic cold forceps biopsy. Specimens will be stored in formalin. Specimens for HPV genotyping will be processed by the Atila Biosystems. Specimens for the tissue bank will be directed to the biorepository owned by Dr. Cornelia Trimble, where they will be processed and cryopreserved for analysis in future studies.

# 8.3.8 HRA, BIOPSY, AND SURGICAL ABLATION

At weeks 16 and 28, if there is residual disease visualized by HRA, patients will undergo repeat HRA with surgical ablation in the operating room by fulguration. If not obtained in the HRA in the office prior to ablation in the operating room, biopsies will be obtained prior to fulguration and resection specimens will be taken fresh to surgical pathology. If possible, a frozen section will be performed to confirm the presence or absence of AIN2/3. The frozen section block will be banked at –70°C in Dr. Cornelia Trimble’s biorepository at Johns Hopkins.

## Compensation

Study subjects will receive a total of $200 if they participate in the entire study. A check will be mailed to their home within a month after each visit. Study subjects will receive $20 for visits at weeks -4, 0, 2, 4, 16, 28, 41 and the postop visit and $40 for the visit at week 16.

# MEASUREMENT OF EFFECT

Although the clinical benefit of these drug(s) has not yet been established, the intent of offering this treatment is to provide a possible therapeutic benefit, and thus the patient will be carefully monitored for AIN regression, HPV viral presence/absence through genotyping and symptom relief in addition to safety and tolerability. **Patients undergoing anal cytology and HRA evaluation will be receiving standard of care surveillance through these surveillance strategies.**

- - 1. Methods for Evaluation of Measurable Disease
- **Anal cytology and HRA**: determine regression from AIN III to AIN I or no evidence of AIN.
- **HPV viral genotyping**: determine efficacy of Artesunate suppository by the eradication of HPV.

# DATA REPORTING / REGULATORY REQUIREMENTS

Adverse event lists, guidelines, and instructions for AE reporting can be found in Section 6.0 (Adverse Events: List and Reporting Requirements).

## Data Reporting

The frequency and severity of all toxicities will be tabulated from CRFs and summarized for review in a descriptive fashion. All CRFs will be reviewed by the principal investigator on a weekly basis with the research team. The initial review of toxicity will be completed after the first treatment group has completed the intervention portion of the study.

Data collected at study visits at both institutions will be entered into the study REDCap database housed at Johns Hopkins.

The secondary efficacy endpoints will be compared with analogous data obtained in an open prospective database, “Development of a Prospective HRA Database” (Johns Hopkins IRB# [NA_00078993](https://e-irb.jhmi.edu/eirb2/Rooms/DisplayPages/LayoutInitial?Container=com.webridge.entity.Entity%5BOID%5B1E381BBFC925834D936BB3B006F85B94%5D%5D) ). This observational cohort protocol has been open since 2012 and includes over 250 subjects. These subjects are all of the patients that have been treated with HRA and ablation in the Johns Hopkins HRA Clinic. We will compare changes in HPV viral load and AIN pathology from baseline to HRA evaluation at 16, 28, and 40 weeks.

**10.2 Risks to privacy of individuals, confidentiality of data**

There is a potential risk of loss of confidentiality. To ensure that all information and specimens collection remain confidential and anonymous, all study data will be de-identified. Only the research team will have access to study participant private data. All study study specimens are de-identified and cannot be linked to the subjects. Specimens will be barcoded before storage so that patient identifiers will not be available to the laboratories performing the assays proposed in this application.

To ensure that all study data remains confidential, all study data will be maintained on an encrypted password-protected database. Patient identifying information will not be associated with the study research data. Only the research investigators will be able to access a list connecting patient identifiers with the study ID, and this can only be provided upon special request from the study database managers. Electronic research charts and regulatory documentation are maintained in an eBinders system hosted by Florence Health Systems under a Business Associate Agreement (BAA) between Johns Hopkins University and Florence. The eBinder Suite is compliant with 21CFR Part 11, HIPAA/the Privacy Rule, and 12CFR part 31 and 812.

# Subject completion/dropout

## Definition of a Dropout

A dropout is defined as any subject who did not come back for at least the week 16 visit as described in the protocol. A subject who returns for the concluding visit foreseen in the protocol is considered to have completed the study participation. Dropouts will be replaced to reach the target enrollment.

## Procedures for Handling Dropouts

The investigator will make a written attempt to contact those subjects who do not return for scheduled visits or follow-up. Information gathered should be described on the Study Conclusion page of the CRF.

## Reasons for Dropout

It should be specified on the Study Conclusion page of the case report form which of the following possible reasons were responsible for dropout of the subject from the study:

- serious adverse event
- non-serious adverse event
- Protocol violation (specify)
- Consent withdrawal, not due to an adverse event
- Migration from the study area
- Lost to follow-up
- Other (specify)

# CLINICAL MONITORING

| The SKCCC Compliance Monitoring Program will provide external monitoring for JHU-affiliated sites in accordance with SKCCC DSMP (Version 6.0, 02/21/2019). The SMC Subcommittee will determine the level of patient safety risk and level/frequency of monitoring. |
| --- |

At University of Wisconsin Carbone Cancer Center (UWCCC) quality assurance and monitoring activities are determined by study sponsorship and risk level of the protocol as determined by the PRMC. All protocols (including Intervention Trials, Non-Intervention Trials, Behavioral and Nutritional Studies, and trials conducted under a Training Grant) are evaluated by the PRMC at the time of committee review. This protocol will be subject to standard monitoring. This means that there will be a review of subject safety at regularly scheduled staff meetings where the results of each subject’s treatment are discussed. The discussion includes the number of subjects enrolled, significant toxicities, dose adjustments, and responses observed. Protocol Summary Reports are submitted on an annual basis by the study team for review by the DSMC. This study will be subject to compliance with the UWCCC Data and Safety Monitoring Plan (version 02/13/2017. Data will be reviewed by Johns Hopkins SMC committee as well as UWCCC compliance team will perform Quality Assurance Reviews and Internal Audits on an annual basis.

# ETHICS AND REGULATORY CONSIDERATIONS

**13.1 Institutional Review Board**

This protocol and an eIRB application and any other required documents will be submitted to the Johns Hopkins IRB and CRO, and their written unconditional approval will be in the possession of the investigator before commencement of the study protocol. No deviations from, or changes to, the protocol will be initiated without prior written IRB approval. The IRB/CRO will be informed of all protocol violations, informed consent changes, or revisions of other documents originally submitted for review. The IRB/CRO will also be informed of serious adverse events occurring during the study, all subsequent protocol modifications, or new information that may affect adversely the safety of the subjects or the conduct of the study. Annual updates will be submitted.

The JHM IRB will be the single IRB of record for this study. A reliance agreement will be fully executed between the Johns Hopkins IRB and the University of Wisconsin, Madison IRB for this protocol.

**13.2 Informed consent**

Information should be given in both oral and written form whenever possible. Subjects will be given ample opportunity to ask about details of the study. Subjects will be informed about the expected risks and benefits, and procedures of the study protocol.

They will be informed of alternative procedures. The informed consent form will be approved by the JHM IRB for both sites. Written consent will be obtained before screening for study enrollment.

Revisions to the informed consent process are being permanently adopted for this research due to the COVID19 pandemic. Depending on institutional and local guidelines, the consenting process can be done in person or via telemedicine visit. Teleconsent will be used as opposed to in person consenting where possible to reduce unnecessary in person encounters specifically for a consent procedure. In the event teleconsent is utilized, participants will be provided with a copy of the Informed Consent prior to the teleconsent meeting either via email, fax, mail or previously provided during an in person visit.

Participants will be given adequate time to consider the research study and ask questions prior to signing the consent form. The consent designee must verify the participant physically signed the consent document either by viewing via video conference, obtaining a photo of the signed consent document; or obtaining verbal confirmation from the participant that he/she signed the consent form or agreed to participate electronically. The participant or LAR will sign and date/time the informed consent document. The document is then mailed, emailed or faxed to the consent designee. The participant will be asked to return the original signed document during their first in person visit. If the Informed Consent form is mailed to the consent designee by the participant the IRB-approved consent designee will sign the copy, which they possess after the participant has acknowledged signature on their copy. Once the original is received by the consent designee the copies will be attached to make a single document. In all other instances, once received, the IRB-approved consent designee signs, dates/times the informed consent document.

At the time of the first clinical encounter post teleconsent, where physician/mid-level provider consent is required, the physician/mid-level provider will review any additional study participant questions and discuss the risks, benefits and alternatives of the study in full detail, completing the physician/mid-level component of the consent process. If physician/Mid-level provider consent occurs remotely in a separate encounter from the main consent process, the previously stated process must be followed for the entire consent conversation. The Documentation of Physician Consent Form is then signed, dated/timed and all components of the consent are combined to one document. After the Informed Consent process is completed, the IRB approved study team member will follow institutional guidelines for uploading research consents to the electronic medical record system, including a note confirming the consent process. The entire consent document is also then filed in the research record.

Once patients sign consent, will be assigned a study ID, and their demographic and clinical information will be entered in a web-based, JHED-authenticated database. This information is also entered in the institutional database CRMS. To maintain confidentiality, the study-specific ID will be used to label all specimens collected and banked during the study. Only the clinical team and program manager will have access to PHI. All other members of the research team will only have access to study-specific IDs.

# STATISTICAL CONSIDERATIONS

## Study Design/Endpoints

The primary objective of this trial is to determine the MTD or recommended Phase 2 dose (RP2D) of Artesunate. The MTD will be determined using a modified 3 + 3 design, in which the MTD is defined as the highest dose level tested at which ≤20% patients experience dose limiting toxicity (DLT). The 3 + 3 dose escalation will consist of 6 dose levels in combination with variation in dosing schedules the single agent Artesunate. In addition, if DLT’s are identified at any dose, there are possible intermediate dose de-escalation steps allowed (i.e. to have only one administration at a particular dose) An expansion cohort will occur at MTD. Once MTD is found, then secondary outcomes will be evaluated. There is a 1-week washout period before advancing to the next Artesunate dose level.

| **Dose Escalation Design** | | | | |
| --- | --- | --- | --- | --- |
| **Cohort** | **Dose (mg)** | **Number of Treatment Cycles** | **Designated Weeks of Artesunate Administration** | **Number of patients** |
| **I** | 200 | 2 | 0, 2 | 3 |
| **II** | 200 | 3 | 0, 2, 4 | 3 |
| **III** | 400 | 2 | 0, 2 | 3 |
| **IV** | 400 | 3 | 0, 2, 4 | 3 |
| **V** | 600 | 2 | 0, 2 | 3 |
| **VI** | 600 | 3 | 0, 2, 4 | 3 |

More specifically:

This is a modified 3 + 3 design. Based on previous studies of rectally administered Artesunate for the treatment of malaria, the safety profile demonstrates a low probability of dose limiting toxicity in children, as well as adults. The first cohort will consist of 3 patients who receive the initial dose of a 200-mg suppository administered in cycles of 5 daily doses at weeks 0 and 2. If none of the three patients in a cohort experiences a dose-limiting toxicity, another three patients will be treated at the next higher dose level. If 1 out of 3 patients experiences dose-limiting toxicity, then the next 3 patients will be treated at the same dose level. If 2 out of 6 patients experience a DLT, then de-escalation to the next lower level will occur. The modification of the 3 + 3 design consists of the extra de-escalation steps as noted on the left of the diagram pictured below.

If any dose has 2 (or more) DLTs then the MTD will be the dose that is below that dose that has 1 or less DLTs out of 6 patients. Once the MTD is identified then additional patients will be added to this cohort to allow a total of 12 patients.

**Cohort I**:

**200 mg** x 5 daily doses/cycle.

**2 cycles** administered at weeks 0, 2

**Cohort III**:

**400 mg** x 5 daily doses/cycle.

**2 cycles** administered at weeks 0, 2

**Cohort IV**:

**400 mg** x 5 daily doses/cycle.

**3 cycles** administered at weeks 0, 2, 4

**De-escalate** to **200 mg** x 5 daily doses/cycle.

**1 cycle** administered at week 0

**De-escalate** to **400 mg** x 5 daily doses/cycle.

**1 cycle** administered at week 0

**Cohort II**:

**200 mg** x 5 daily doses/cycle.

**3 cycles** administered at weeks 0, 2, 4

**Cohort VI**:

**600 mg** x 5 daily doses/cycle.

**3 cycles** administered at weeks 0, 2, 4

**Cohort V**:

**600 mg** x 5 daily doses/cycle.

**2 cycles** administered at weeks 0, 2

**De-escalate** to **600 mg** x 5 daily doses/cycle.

**1 cycle** administered at week 0

**At MTD, expansion cohort**, n = 12.

De-escalate

De-escalate

De-escalate

De-escalate

## Sample Size/Accrual Rate

A total of up to 60 patients (36 expected from the 3+3 design, with extra allowable with the modified escalation schema to account for de-escalation in cycle frequency) will be accrued to the study. It is expected that 6 evaluable patients will be enrolled per month.

## Analysis of Primary and Secondary Endpoints

- - 1. Analysis of safety endpoints

The NCI common terminology criteria for adverse events (AE) (CTCAE 4.0) will be used to evaluate toxicity. We will consider a toxicity to be an adverse event that is possibly, probably or definitely related to treatment. The maximum grade of toxicity for each category of interest will be recorded for each patient, and the summary results will be tabulated by category, grade, and dose level. Serious (≥ Grade 3) toxicities will be described on a patient-by-patient basis and will include any relevant baseline data.

Once the MTD (or RP2D) is found, then secondary outcomes will be evaluated based on MTD.

- - 1. Analysis of secondary efficacy endpoints

Regression of AIN 2/3 to either AIN 1 or the absence of AIN will be used as dichotomous variables and the proportion of patients with the characteristics and the corresponding 95% confidence intervals will be calculated.

HPV viral genotype will be collected longitudinally over time as a qualitative measure.

# REFERENCES

1. Joseph DA, Miller JW, Wu X, et al. Understanding the burden of human papillomavirus-associated anal cancers in the US. Cancer 2008;113:2892-900.

2. Goldstone SE, Hundert JS, Huyett JW. Infrared coagulator ablation of high-grade anal squamous intraepithelial lesions in HIV-negative males who have sex with males. Dis Colon Rectum 2007;50:565-75.

3. Artesunate Rectal Suppository World Heath Organization Global Health Malaria Program. 2010 at http://www.fda.gov/downloads/advisorycommittees/committeesmeetingmaterials/drugs/anti-infectivedrugsadvisorycommittee/ucm209921.pdf.)

4. Hien TT, White NJ. Qinghaosu. Lancet 1993;341:603-8.

5. Disbrow GL, Baege AC, Kierpiec KA, et al. Dihydroartemisinin is cytotoxic to papillomavirus-expressing epithelial cells in vitro and in vivo. Cancer Res 2005;65:10854-61.

6. Siegel EM, Patel N, Lu B, et al. Circulating biomarkers of iron storage and clearance of incident human papillomavirus infection. Cancer Epidemiol Biomarkers Prev 2012;21:859-65.

7. Batty KT, Thu LT, Davis TM, et al. A pharmacokinetic and pharmacodynamic study of intravenous vs oral artesunate in uncomplicated falciparum malaria. Br J Clin Pharmacol 1998;45:123-9.

8. O'Neill PM, Posner GH. A medicinal chemistry perspective on artemisinin and related endoperoxides. J Med Chem 2004;47:2945-64.

9. National survey shows HPV vaccine rates rail other teen vaccines. at www.cdc.gov/media/releases/2011/p0825_hpv_vaccine.html.)

10. Richel O, de Vries HJ, van Noesel CJ, Dijkgraaf MG, Prins JM. Comparison of imiquimod, topical fluorouracil, and electrocautery for the treatment of anal intraepithelial neoplasia in HIV-positive men who have sex with men: an open-label, randomised controlled trial. Lancet Oncol 2013;14:346-53.

11. Snyder SM, Siekas L, Aboulafia DM. Initial Experience with Topical Fluorouracil for Treatment of HIV-Associated Anal Intraepithelial Neoplasia. J Int Assoc Physicians AIDS Care (Chic) 2011;10:83-8.

12. Kwan TT, Lo SS, Tam KF, Chan KK, Ngan HY. Assessment of knowledge and stigmatizing attitudes related to human papillomavirus among Hong Kong Chinese healthcare providers. Int J Gynaecol Obstet 2012;116:52-6.

13. Meshnick SR. Artemisinin: mechanisms of action, resistance and toxicity. Int J Parasitol 2002;32:1655-60.

14. World Health Organization. Artemisinin Derivatives: Summary of Nonclinical Safety Data Introductory Remarks. 2002. 2002. at http://apps.who.int/prequal/info_applicants/Guidelines/Nonclinical_Overview_Artemisinin-Derivatives.pdf.)

15. FDA Briefing Document for the Anti-Infective Drugs Advisory Committee. Artesunate Rectal Suppository. World Health Organization Global Health Malaria Program. at http://www.fda.gov/downloads/advisorycommittees/committeesmeetingmaterials/drugs/anti-infectivedrugsadvisorycommittee/ucm209921.pdf.)

16. Lin KY, Guarnieri FG, Staveley-O'Carroll KF, et al. Treatment of established tumors with a novel vaccine that enhances major histocompatibility class II presentation of tumor antigen. Cancer Res 1996;56:21-6.

17. Efferth T, Dunstan H, Sauerbrey A, Miyachi H, Chitambar CR. The anti-malarial artesunate is also active against cancer. Int J Oncol 2001;18:767-73.

18. Galal AM, Ross SA, ElSohly MA, et al. Deoxyartemisinin derivatives from photooxygenation of anhydrodeoxydihydroartemisinin and their cytotoxic evaluation. J Nat Prod 2002;65:184-8.

19. Sertel S, Eichhorn T, Sieber S, et al. Factors determining sensitivity or resistance of tumor cell lines towards artesunate. Chem Biol Interact 2010;185:42-52.

20. Novartis, Anti-Infective Drugs Advisory Committee Meeting. Coartem (artemetherlumefantrine) Tablets for the treatement of malaria in patients with acute, uncomplicated infections due to Plasmodium falciparum or mixed infections including P. falciparum. NDA 22-268. 2008. at http://www.fda.gov/ohrms/dockets/ac/08/briefing/2008-4388b1-02-Novartis.pdf.)

21. Bohling SD, Allison KH. Immunosuppressive regulatory T cells are associated with aggressive breast cancer phenotypes: a potential therapeutic target. Mod Pathol 2008;21:1527-32.

22. Krishna S, Bustamante L, Haynes RK, Staines HM. Artemisinins: their growing importance in medicine. Trends Pharmacol Sci 2008;29:520-7.

23. Anfosso L, Efferth T, Albini A, Pfeffer U. Microarray expression profiles of angiogenesis-related genes predict tumor cell response to artemisinins. Pharmacogenomics J 2006;6:269-78.

24. Mizuuchi H, Kudo R, Tamura H, et al. Identification of transferrin receptor in cervical and endometrial tissues. Gynecol Oncol 1988;31:292-300.

25. Zandberg DP, Bhargava R, Badin S, Cullen KJ. The role of human papillomavirus in nongenital cancers. CA Cancer J Clin 2013;63:57-81.

26. Efferth T, Kaina B. Toxicity of the antimalarial artemisinin and its dervatives. Crit Rev Toxicol 2010;40:405-21.

27. Nontprasert A, Pukrittayakamee S, Dondorp AM, Clemens R, Looareesuwan S, White NJ. Neuropathologic toxicity of artemisinin derivatives in a mouse model. Am J Trop Med Hyg 2002;67:423-9.

# APPENDIX A: STUDY CALENDAR

Baseline evaluations are to be conducted within 4 week prior to start of protocol therapy. In the event that the patient’s condition is deteriorating, laboratory evaluations should be performed per they study physician’s discretion within 48 hours prior to initiation of the next cycle of therapy.

| **Surveillance Period** | | | | | | | | |
| --- | --- | --- | --- | --- | --- | --- | --- | --- |
|  | Screen | Cycle 1 (wk 0) | Cycle 2 (wk 2)^1^ | Cycle 3 (wk 4)^1^ | Wk 6 | Wk 16 | Wk 28 | Wk 40 |
| Artesunate |  | X | X | +/- |  |  |  |  |
| Informed consent | X |  |  |  |  |  |  |  |
| Demographics | X |  |  |  |  |  |  |  |
| Complete History | X |  |  |  |  |  |  |  |
| Physical exam | X |  |  |  |  |  |  |  |
| Vitals signs | X | X | X^2^ | X^2^ | X | X | X | X |
| Urine hCG (if female) | X | X | X^3^ | X^3^ |  |  |  |  |
| HIV test with reflex CD4 count | X |  |  |  |  |  |  |  |
| AE diary cards |  | X |  |  |  |  |  |  |
| Anal pap | X |  |  |  |  | X | X | X |
| Anal brushes | X | X | X^2^ | X^2^ | X | X | X | X |
| Anoscopy | X |  | X^2^ | X^2^ | X | X | X | X |
| HRA | X |  |  |  |  | X | X | X |
| HPV testing | X |  |  |  | X | X | X | X |
| Bloods for banking | X |  |  |  | X | X | X | X |

+/-: The patient may or may not get the Artesunate or undergo anal pap test depending on the dosing schedule they are assigned to.

***Please note that anoscopy is part of the HRA evaluation. Anoscopy without concurrent HRA will be used to evaluate for any toxicity to the local tissue at the dentate line at the week 6 visit.

****Participants who are on the 2 cycle dosing schedule will still be seen on cycle 3 week 4 but will not have a urine hCG test or get dispensed Artesunate.

^1^may be done as telemedicine visits

^2^will be obtained at in-person visits only

^3^for telemedicine dosing visits, the patient will do a home pregnancy test and will send a picture to the study team, along with an attestation of negative pregnancy test.

APPENDIX B: Artesunate Intra-anal Suppository Symptom Diary

Study ID:_________________

Cycle # (circle one) 1 2 3 Treatment cohort (choose one):

**Artesunate, 200mg**

**Artesunate, 400 mg**  **Artesunate, 600 mg**

**Total Cycles planned: 1 2 3**

Please record any symptoms you experience every day. If you miss a day and can recall what it looked like and how it felt you may add the missing information. If you miss writing in your diary for more than 2 days, do not fill in the missed information, and start recording again on the day that you missed. If you have questions or concerns call your research nurse, or your study coordinator, at ____________________________________

| **Days**  **after first dosing of present cycle** | **Date** | **Suppository taken?** | **Anal pain/**  **ulcer** | **Pain in Abdomen** | **Nausea/**  **Vomiting** | **Headache** | **Dizziness** | **No problems** | **Other problems (list)** |
| --- | --- | --- | --- | --- | --- | --- | --- | --- | --- |
| 0 |  |  |  |  |  |  |  |  |  |
| 1 |  |  |  |  |  |  |  |  |  |
| 2 |  |  |  |  |  |  |  |  |  |
| 3 |  |  |  |  |  |  |  |  |  |
| 4 |  |  |  |  |  |  |  |  |  |
| 5 |  |  |  |  |  |  |  |  |  |
| 6 |  |  |  |  |  |  |  |  |  |
| 7 |  |  |  |  |  |  |  |  |  |
| 8 |  |  |  |  |  |  |  |  |  |
| 9 |  |  |  |  |  |  |  |  |  |
| 10 |  |  |  |  |  |  |  |  |  |
| 11 |  |  |  |  |  |  |  |  |  |
| 12 |  |  |  |  |  |  |  |  |  |
| 13 |  |  |  |  |  |  |  |  |  |
| 14 |  |  |  |  |  |  |  |  |  |
| Reviewed by: | | | | | | Date: | | | |
